# Thank you to the reviewers of Rheumatology Advances in Practice 2024

**DOI:** 10.1093/rap/rkaf002

**Published:** 2025-02-13

**Authors:** Ai Lyn Tan

**Affiliations:** NIHR Leeds Biomedical Research Centre, Leeds Teaching Hospitals NHS Trust, Leeds, UK; Leeds Institute of Rheumatic and Musculoskeletal Medicine, University of Leeds, Leeds, UK

Rheumatology Advances in Practice (RAP) continues to go from strength to strength. I am filled with pride and gratitude for what we have accomplished together as a team, as I reflect on my tenure as the Editor-in-Chief of the journal. The success of the journal is testament to the collaboration and creativity of every member of the RAP family, always empowered to go a step further to extend the scope of the journal. It has been a tremendous privilege to serve in this role, and as I prepare to hand over the reins, I am appreciative of the kindness of the team that forms the foundation of the journal’s forward vision.

First and foremost, I extend my deepest thanks to our reviewers. Your expertise, diligence and generosity have been the cornerstone of our journal’s success. In 2024 alone, you contributed many valuable hours to ensure the rigor and relevance of our publications. Your thoughtful insights and constructive feedback not only elevated the quality of the work we publish, but also guided and inspired our authors. We celebrate and thank you for your essential role in advancing rheumatology.

In the past year, we have continued to see the journal flourish as a trusted platform for disseminating high-quality research in rheumatology. We are proud of our popular Current and Future Advances in Practice series [1], which delves into topics and knowledge in rheumatology that are underserved and therefore are very much welcomed by our readers. RAP continues to ensure it is aligned with the current interests of our readers. Therefore, in the next year, I am excited to announce that we will have a special issue on artificial intelligence in rheumatology that aims to enhance our readers’ knowledge.

At RAP, we believe in being as inclusive as possible—one of the values of the British Society for Rheumatology [2]. In recent years we added lay summaries to our articles to benefit our non-specialist readers [3]. In 2024, we continued with our ambition to support inclusivity, so we expanded our social media presence and we are now on LinkedIn [4] in order to increase our reach into the community. In collaboration with our associate editors, we developed a mentoring scheme for reviewers, focusing on encouraging and nurturing early career researchers, to improve their peer review skills and help them to gain an understanding of academic publishing and the editorial process [5].

Our journal’s growth would not have been possible without the exceptional team at Oxford University Press. From managing the complexities of publication to ensuring the accessibility and visibility of our work, the professionalism has been extraordinary. To our dedicated editorial board and the editorial team, I am profoundly grateful for the wisdom, collaboration and unwavering support. Together, we have championed initiatives that broaden the journal’s reach and impact. RAP truly embodies a close knit family in which every action is carefully harmonized for the greater mission of improving outcomes for patients.

As I step down, I am delighted to welcome my successor, Dr Mwidimi Ndosi, as the new Editor-in-Chief. Mwidimi is a widely respected leader in rheumatology and has been a dedicated co-editor for RAP. I am confident that under his stewardship, the journal will continue to grow and thrive, setting new benchmarks for excellence.

To our authors, readers and the global rheumatology community, thank you for trusting us to share your work and for engaging with the knowledge and ideas that shape our field. To the patients, thank you for focusing our attention on what is important in rheumatology. The journey ahead is bright and I look forward to seeing how Rheumatology Advances in Practice continues to evolve and inspire.

## Data Availability

No new data were generated or analyzed in support of this article.

## List of 2024 reviewers

Abhishek Abhishek

Anne-Catherine Dens

Ade Adebajo

Adina Tschuppert

Ai Li Yeo

Ai Lyn Tan

Ailish Byrne

Akira Yoshida

Alain Lescoat

Alain Saraux

Alan Hakim

Alan T. Kaell

Alejandro Rodriguez-Ruiz

Alessandra Soriano

Alexandra Mundell

Ali Jawad

Alvaro Gomez

Alwin Sebastian

Ana Rita Cruz-Machado

Ana Teodósio Chícharo

Anastasia-Vasiliki Madenidou

André Lucas Ribeiro

Andrea Di Matteo

Andreas Kronbichler

Andriko Palmowski

Ann K. Rosenthal

Anna Olewicz

Antoni Chan

Antonietta Gigante

Arshid B. Bhat

Asad Zoma

Azmad Kareem B. Anwardeen

Bart Spaetgens

Benjamin Chaigne

Bharatendu Chandra

Brian M. Feldman

Burkhard Möller

Caifeng Li

Carlo Selmi

Carmen Berrocal

Caroline Feldthusen

Caroline V. Cotton

Catherine L. Hill

Charlotte A. Sharp

Chee-Seng Yee

Chen Li

Chih-Wei Tseng

Christopher Sjöwall

Christopher W. Joyce

Ciro Manzo

Claire E. Owen

Clara Marie Elbæk Mistegård

Clifton O. Bingham III

Clio Ribbens

Corinne Fisher

Cristiana Sieiro Santos

Da-Li Tong

Dale Fournier

Dalia Ludwig

Dan P. Kelleher

Daniel Bromage

David Liew

David Beck

David Hughes

David Wong

Deepak Nagra

Delphine Bertrand

Deniz Bayraktar

Dennis McGonagle

Desh Nepal

Didem Saygin

Diederik De Cock

Diego Benavent

Doskaliuk Bohdana

Durga P. Misra

Elaine Dennison

Eleana Ntatsaki

Eleonoora Hintsa Figueiredo

Elizabeth Price

Elliott Lever

Emre Bilgin

Enrico De Lorenzis

Eric Gracey

Ernest H. Choy

Evelien Gielen

Fabian Proft

Fernando Sommerfleck

Fiona A. Pearce

Frances Mills-Baker

Francisco Ortiz-Sanjuán

Gabriel P. Esteves

Gary J. Macfarlane

George E Fragoulis

Giuseppe Lopalco

Gizem Ayan

Gordon J. Hendry

Gregory Booth

Gülnihal Deniz

Gwenda Simons

Hammad Ali

Hazan Karadeniz

Heather A. Rostron

Helen Parsons

Hema Chaplin

Hubert de Boysson

Hubert Marotte

Ilke Coskun Benlidayi

Irem Tasci

Isabela Borges

Ismihan Sunar

Israel Adandom

J. J. Rasker

J. Kennedy Amaral

Jade Skeates

James Griffin

James B. Galloway

Jan Leipe

Jan René Nkeck

Jean-Baptiste Vulsteke

Jill Halstead-Rastrick

Jing-fen Ye

Joe G. Eddison

Jonathan Packham

Julie Begum

Kanon Jatuworapruk

Karin Melsens

Katie Mageean

Katie L. Hackett

Kavita Makan

Keegan P. Knittle

Kunal Chandwar

LaKrista Morton

Latika Gupta

Laura Andreoli

Laure Gossec

Lene Terslev

Lie Dai

Lien Moreel

Lin Tang

Louise Kedroff

Louise Warburton

Lucy R. Wedderburn

Mandeep Sekhon

Manouk de Hooge

Manuel F. Ugarte-Gil

Marc Scherlinger

Marco Di Battista

Margot Van Mechelen

Maria Llop

María Carmen Garcia-Gomez

Marie Vanthuyne

Marie Vermant

Mark J. Garton

Mark M. Bakker

Mark S. Lillicrap

Marwan Bukhari

Masaru Kato

Mats Junek

Maura McCarron

Maurizio Cutolo

Mauryn Oganga

Mayka Freire-Dapena

Md Yuzaiful Md Yusof

Megan Rutter

Meghna Jani

Michaël Doumen

Miguel Angel González-Gay

Mohammad Khinagir

Mohammad Negaresh

Mohit Goyal

Muhammad S. N. Soyfoo

Mwidimi Ndosi

Natalie McCormick

Natasha Cox

Neelam Hassan

Neerinckx Barbara

Nicholas Fuggle

Nicola J. Goodson

Norelee Kennedy

Onengiya Harry

Owen Cronin

Pankti Mehta

Patrick Verschueren

Philip S. Helliwell

Piercarlo Sarzi-Puttini

Pieter Bourgeois

R. Naveen

Rabia Miray Kisla Ekinci

Rachel Black

Raj Amarnani

Raj Sengupta

Raphael Micheroli

Rashmi Roongta

Raziye Burcu Güven Bilgin

Rene Westhovens

Richard Conway

Richard Jones

Richard A. Watts

Robert Biesen

Rona Smith

Roxanne Cooksey

Rucsandra Dobrota

Saion Chatterjee

Samantha L. Hider

Sampada Bhide

Sander van Leuven

Sarah Dyball

Sarah L. Mackie

Sean McKenna

Sebastián Udry

Sebastian E. Sattui

Seyran Naghdi

Shalini Janagan

Sham Santhanam

Sheilla Achieng

Shih-Yang Chen

Shounak Ghosh

Shuayb Elkhalifa

Simon Chatfield

Simone Parisi

Sravan Kumar Appani

Sriharsha Grevich

Stephanie Lembke

Stephanie Mathew

Sytske Anne Bergstra

Takashi Kida

Tayyeba Khursheed

Thomas Hügle

Titilola Falasinnu

Torsten Diekhoff

Tsuneo Sasai

Tsuneyasu Yoshida

Ulf Sundin

Uta Kiltz

Valeria Giorgi

Vanessa Smith

Varvara Choida

Veit Krenn

Victoria A. Flower

Vincent Goeb

Vincenzo Venerito

Vinod Ravindran

Vishal Kakkar

Vivekanand Tiwari

Wesam Gouda

William Tillett

William G. Dixon

William J. Gregory

Wilson Bautista-Molano

Xenofon Baraliakos

Xin-Yue Xiao

Yehuda Shoenfeld

Yeliz Prior

Yen T. Chen

Yongpeng Ge

Yuanyuan Wang

Yunyun Fei

Yves Piette

Zijing Yang

## References

[1] Current and future advances in practice. https://academic.oup.com/rheumap/pages/current-and-future-advances-in-practice (8 December 2024, date last accessed).

[2] British Society for Rheumatology. Vision, mission and values. https://www.rheumatology.org.uk/about-bsr/vision-mission-and-values (8 December 2024, date last accessed).

[3] Tan AL. Thank you to the reviewers of Rheumatology Advances in Practice 2022. Rheumatol Adv Pract 2022;7:rkad013.

[4] LinkedIn. RheumJnl. https://www.linkedin.com/company/rheumjnl/ (8 December 2024, date last accessed).

[5] British Society for Rheumatology. Mentoring scheme – Rheumatology Advances in Practice. https://www.rheumatology.org.uk/news/details/Mentoring-scheme-Rheumatology-Advances-in-Practice (8 December 2024, date last accessed).

